# The genus *Alaolacon* Candèze, a senior synonym of the genus *Eumoeus* Candèze (Coleoptera, Elateridae, Agrypninae)

**DOI:** 10.3897/zookeys.656.8914

**Published:** 2017-02-14

**Authors:** Kôichi Arimoto, Hisayuki Arimoto

**Affiliations:** 1 Entomological Laboratory, Graduate School of Bioresource and Bioenvironmental Sciences, Kyushu University, Fukuoka, 812–8581 Japan; 2 Tedukayama Nishi 3-4-21, Osaka, 812-0053 Japan

**Keywords:** *Eumaeus*, Hemirhipini, *Luzonicus*, new species, Oriental region, replacement name, taxonomy, *Tharopsides*

## Abstract

*Alaolacon* Candèze, 1865 is found to be a senior synonym of *Eumoeus* Candèze, 1874, *Luzonicus* Fleutiaux, 1916 and *Tharopsides* Fleutiaux, 1918. *Alaolacon* is represented by *Alaolacon bakeri* (Fleutiaux, 1916), **comb. n.**, *Alaolacon candezei* Fleutiaux, 1928, *Alaolacon cyanipennis* Candèze, 1865, *Alaolacon fujiokai* **sp. n.**, *Alaolacon griseus* Candèze, 1874, *Alaolacon megalopus* **sp. n.**, *Alaolacon murrayi* (Candèze, 1874), **comb. n.**, and *Alaolacon philippinensis* **nom. n.** This genus is redescribed based on the descriptions of three species, *Alaolacon candezei*, *Alaolacon fujiokai*, and *Alaolacon megalopus* as well as the examination of the holotypes of *Alaolacon cyanipennis* and *Alaolacon murrayi* comb. n. Males of the genus *Alaolacon* exhibit 12-segmented and biflabellate antennae, and the females exhibit 11-segmented and subpectinate antennae. A key to species is provided.

## Introduction

Candèze (1865) established the monotypic genus *Alaolacon* for *Alaolacon cyanipennis* from the Peninsular Malaysia. *Alaolacon griseus* Candèze, 1874 from Thailand and *Alaolacon candezei* Fleutiaux, 1928 from Banggi Island, Malaysia (near Borneo) were described later. All specimens described in this genus were females, with 11-segmented and subpectinate antennae, and the males were undescribed. Candèze (1874) established genus *Eumoeus* for one species, *Eumoeus murrayi*, from India from a male with 12-segmented and biflabellate antennae. He argued that *Eumoeus* was similar to *Alaolacon*, although they had extremely different antennae, and suggested that *Alaolacon* should be combined with *Eumoeus* if its male had biflabellate antennae.

Fleutiaux (1916) established *Luzonicus*, containing only *Luzonicus bakeri* from the Philippines, from a female specimen with 11-segmented and moniliform antennae. Fleutiaux (1918) later established *Tharopsides* including two species, *Tharopsides harmandi* and *Tharopsides bakeri* from Thailand and the Philippines respectively, from males possessing 12-segmented and biflabellate antennae. Fleutiaux (1940) stated that *Eumoeus* was a junior homonym of *Eumaeus* Hübner, 1816 of Lepidoptera, and used *Tharopsides* as the replacement name. Fleutiaux (1947) subsequently stated that *Tharopsides* was a junior synonym for *Luzonicus*, and that antennal differences were sexual dimorphism. However, Casari-Chen (1993, 1994) and Casari (2008) treated *Eumoeus* as a valid name and a monotypic genus, making no mention about the treatments of Fleutiaux (1940, 1947). This paper reviews the taxonomy of these four genera of Hemirhipini and of five of eight included species in order to resolve this confusion.

## Materials

Depositories of the type specimens and non-type specimens examined are as follows:

BMNH
 The Natural History Museum, London



MNHN
Muséum national d’Histoire naturelle, Paris (Edmond Fleutiaux collection) 


IRSNB
Institut Royal des Sciences Naturelles de Belgique, Brussels 


ELKU
 Entomological Laboratory, Kyushu University, Fukuoka 


A generic description of *Alaolacon* was made from the study of the type specimens of *Alaolacon cyanipennis* Candèze, 1865, *Alaolacon candezei* Fleutiaux, 1928, *Alaolacon murrayi* (Candèze, 1874) comb. n. (= *Eumoeus murrayi*) and two new species described here. Species descriptions of *Alaolacon cyanipennis* and *Alaolacon murrayi* are not provided as they are adequately described in Casari-Chen (1993).

We could not find the types of two species, *Alaolacon bakeri* (Fleutiaux, 1916), comb. n. (= *Luzonicus bakeri*) and *Alaolacon philippinensis* comb. n. (= *Tharopsides bakeri* Fleutiaux, 1918) in the collections of BMNH, IRSNB nor the MNHN and have not examined these species. It was not possible to prepare a description of *Alaolacon griseus* Candèze, 1874.

## Methods

Photographs of specimens were taken by a single-lens reflex camera (Canon EOS 70D) with a macro lens (Canon macro photo lens MP-E 65mm), and then images taken in a series of focal planes were combined using CombineZM 1.0.0 software (Alan Hadley, United Kingdom). Micrographs were prepared using a scanning electron microscope (SEM: Hitachi S-3000N) without gold coating.

Most structures were observed under a stereo microscope (Olympus-SZX9). Measurements are in millimeters and were made with a micro ruler (MR-2, Kenis Limited, Ôsaka; minimum scale value: 0.05 mm). Specimens were softened in warm water. The pregenital segments and genitalia extracted from the abdomen were soaked in 10% KOH solution (room temperature, male: 2 hours, female: 48 hours). The parts were cleaned in 30% ethanol (10 min) and dehydrated in 99.5% ethanol (5 min) and then mounted in glycerin on microscope slides, except the female genitalia, which were examined in water and then mounted in glycerin. A transmission microscope (Nikon Y-IDT) with a *camera lucida* was used to examine slides and for drawing. Morphological terminology follows Calder (1996), and Casari-Chen (1993) and Costa et al. (2010) in part. Photographs and drawings were edited with image editing software (Adobe Photoshop 7.0).

The following abbreviations are used:

BL
 body length from head to elytral apices 


BW
 the maximum body width 


MIE
 the minimum distance between the eyes 


MAE
 the maximum distance across the eyes 


OI
 Ocular index: MIE/MAE × 100 


PL
 the maximum pronotum length including posterior angles 


PML
 length of the midline of pronotum 


PW
 the maximum pronotum width including posterior angles 


PI
 Pronotam index: PL/PW × 100 


EL
 the maximum elytra length 


EW
 the maximum elytra width 


EI
 Elytra index: EL/EW × 100 


## Taxonomy

### Genus Alaolacon

Taxon classificationAnimaliaColeopteraElateridae

Candèze, 1865

Alaolacon Candèze, 1865: 13 (original description; type species: Alaolacon cyanipennis Candèze, 1865; by monotypy; in Mélanactides); Gemminger and Harold 1869: 1498 (catalogue of Coleoptera); Candèze 1874: 114 (in tribe Alaites); Candèze 1891: 29 (short description; in tribe Alaites), 241 (index); Schwarz 1906: 316 (catalogue); Hyslop 1921: 625 (type species of genera of Elateridae); Schenkling 1925: 40 (catalogue); Fleutiaux 1926: 102 (catalogue); Fleutiaux 1928: 177 (description); Casari-Chen 1993: 227 (description; removed from Hemirhipini); Casari 2008: 166 (key to genera of Hemirhipini; replaced in Hemirhipini).
Eumoeus Candèze, 1874: 113 (original description; type species: Eumoeus murrayi Candèze, 1874; by monotypy; in tribe Alaites), 214 (as “Eumaeus”; index); Candèze 1891: 29 (short description; in tribe Alaites), 243 (index); Schwarz 1906: 32 (key to genera of Hemirhipini), 40 (catalogue); Hyslop 1921: 645 (type species); Schenkling 1925: 51 (as “Eumaeus”; catalogue); Fleutiaux 1928: 178 (as “Eumaeus”; comments); Fleutiaux 1947: 306 (as junior homomym of Eumaeus Hübner, 1816 (Lepidoptera)); Casari-Chen 1993: 241 (description; removed from Hemirhipini); Casari 2008: 164 (key to genera of Hemirhipini; replaced in Hemirhipini) **Syn. n.**
Luzonicus Fleutiaux, 1916: 232 (original description; type species: Luzonicus bakeri Fleutiaux, 1916; by monotypy; in Corymbitinae); Schenkling 1927: 405 (catalogue); Fleutiaux 1947: 306 (key to genera of Oxynopterinae; description); Tarnawski 2001: 306 (catalogue of Ctenicerini, Athoinae).
Tharopsides Fleutiaux, 1918: 235 (original description; type species: Tharopsides harmandi Fleutiaux, 1918); Hyslop 1921: 671 (type species); Fleutiaux 1924: 176 (reprinting of original description); Fleutiaux 1928: 178 (taxonomic comments); Schenkling 1927: 509 (catalogue); Fleutiaux 1940: 40 (as replacement name for Eumoeus Candèze, 1874; in Hemirhipinae); Fleutiaux 1947: 306 (as synonym of Luzonicus Fleutiaux, 1916).

#### Diagnosis.

Setae flat, wider at midlength than base, with longitudinal micro carinae (Figs 36, 37); interspaces between punctures greater than puncture diameter except for narrower interspaces on head and pronotum; supra-antennal carinae not continuous across frons; frontoclypeal region gradually sloping to base of labrum; antennae 12-segmented and biflabellate in male (Figs 23, 42) or 11-segmented and subpectinate in females (Fig. 5); mandibles bidentate; hypomeron with mesal edge with impunctate ridge next to prosternal suture and carinate anterolaterad (Figs 8, 26, 45: arrow), posterior edge with two angles near mid-length (Figs 9, 27, 46: arrows); scutellum widest posteriorly or with parallel sides; elytral intervals convex; hind wings with vein r4 translucent (Figs 11, 29, 48); parameres of male aedeagus not fused and without acute lateral subapical barb (Figs 34, 35, 53, 54).

#### Redescription.

**Figures 1–3.**
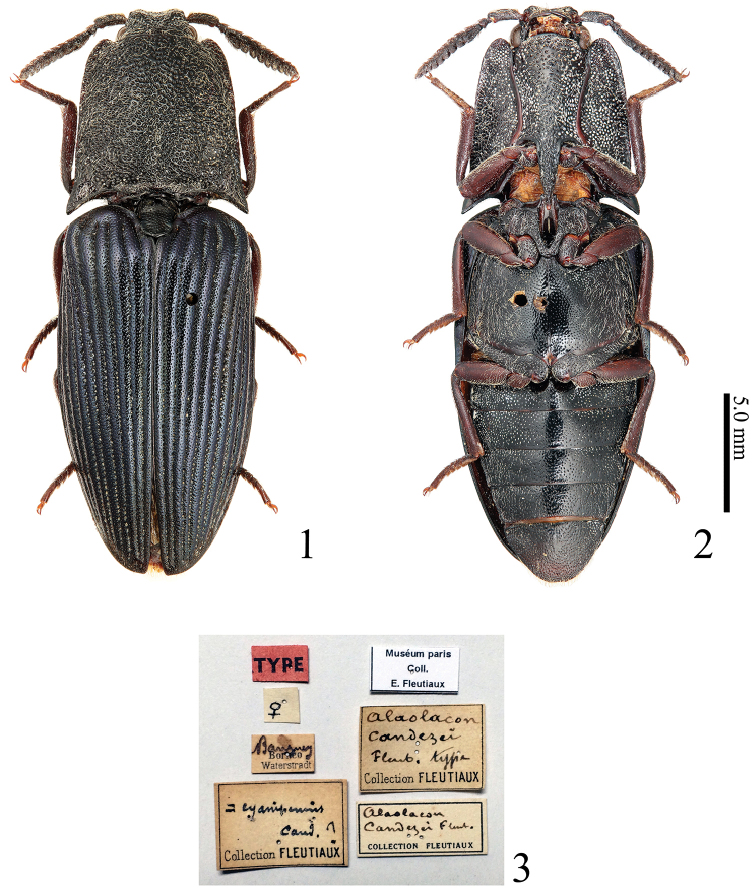
*Alaolacon candezei* Fleutiaux, 1928, female, holotype. **1** habitus, dorsal view **2** ditto, ventral view **3** data labels.

Adult. *Body* (Figs 1, 17, 20, 38, 55) 11–24 mm; surface smooth, with or without metallic luster on elytra; interspaces between punctures greater than puncture diameter except for narrower interspaces on head and pronotum. Vestiture. Setae flat, wider at midlength than base, with longitudinal micro carinae (Fig. 36); carinae converge at apex, apices acute or transverse (Fig. 37). ***Male***. Antennomeres III–XII with setae filiform ventrally.

**Figures 4–11.**
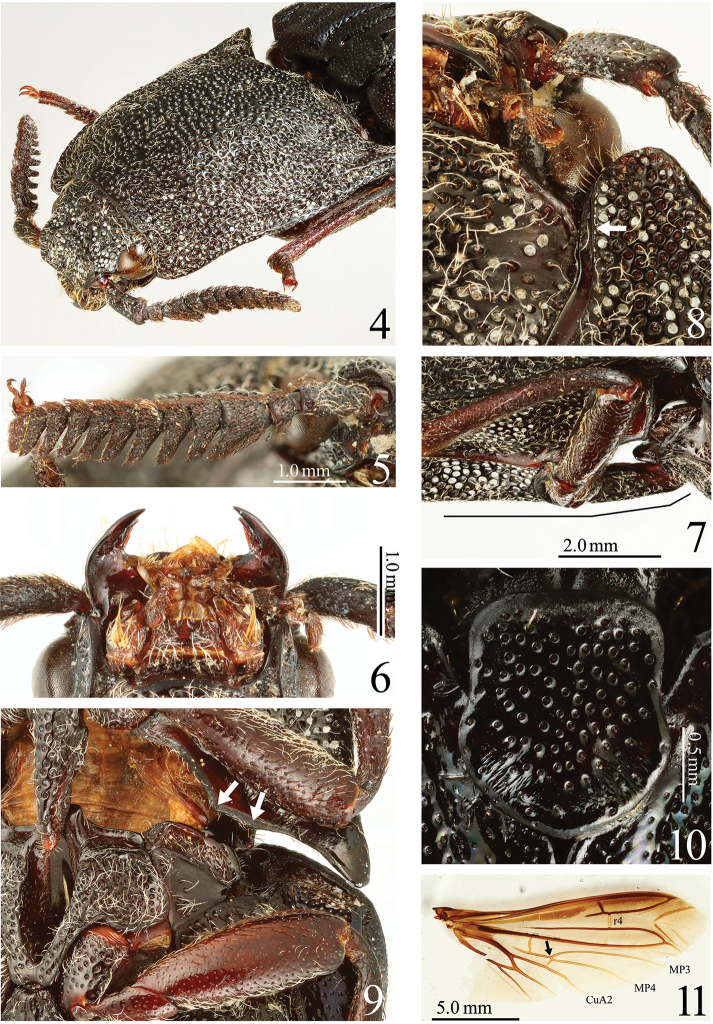
*Alaolacon candezei* Fleutiaux, 1928, female, holotype. **4** head and pronotum, anterolateral view **5** right antenna, anterior side (dotted line: apical half part of antennomere XI thinner than its basal half part) **6** mouth-parts **7** prosternal process, lateral view **8** anterior angle of hypomeron (arrow: mesal edge carinate anterolaterad) **9** posterior part of hypomeron and mesothorax, ventral view (arrows: posterior margin with two angles) **10** scutellum **11** right hind wing (arrow: cross vein between veins MP4 and CuA2 located at contact point between veins MP3 and MP4).

*Head* (Figs 4, 22, 41) depressed longitudinal medially, depression becoming narrow and shallow posteriorly. Frontal depression moderate (Figs 4, 41) to deep (Figs 22, 56). Eyes small to very large (OI: 44–74). Supra-antennal carina not continuous across frons. Frontoclypeal region gradually sloping to base of labrum. Labrum subtrapezoidal; anterior angles rounded. Antennae not reaching pronotum posterior lateral apices; antennomere I cylindrical; antennomere II shortest. ***Male*** (Figs 23, 42). 12-segmented; antennomeres III–XI biflabellate; antennomere XII blade-like. ***Female*** (Fig. 5). 11-segmented; antennomere III subpectinate to trapezoidal, longer than wide (1.2–1.4 × as long as wide); antennomeres IV-X pectinate, shorter than wide (less than 0.6 × as long as wide); antennomere XI elliptical. Mandibles bidentate (Fig. 6). Labium (Figs 6, 24, 43); mentum membranous in anterior part; prementum widest anteriorly, with anterior margin fringed with short setae. Apical maxillary palpomere 1.3–1.8 × as long as wide.

*Prothorax* shorter to longer than wide, widest posteriorly or at mid-length. Pronotum with anterior angle bisinuate (Figs 1, 17, 20, 56) or rounded (Fig. 38); hind angles unicarinate; median longitudinal depression present extending at almost all pronotal length (Figs 4, 22, 56) or at pronotal anterior half (Fig. 41). Hypomeron concave; impunctate posterad; anterior angles rounded (Fig. 8) to acute (Figs 26, 45); external margins of depressions for reception of forelegs not carinate; mesal edge with elevated impunctate ridge next to prosternal suture, carinate anterolaterad (Figs 8, 26, 45: arrow); posterior edge with two angles near midlength (Figs 9, 27, 46: arrows). Prosternum produced forwards, exceeding anterior angles of pronotum; prosternal spine inclined dorsally behind procoxae weakly (at less than 10 degrees, Fig. 7) to strongly (more than 10 degrees, Figs 25, 44).

*Mesothorax*. Scutellum longer than wide; anterior margin straight, well defined by wrinkled band; sides concave or straight, widest posteriorly (Figs 10, 28, 56) or parallel (Fig. 47); rounded posterad. Mesosternum and metasternum not fused. Mesosternal cavity with median shiny band formed by dense yellow setae (Fig. 2, 21, 39). Mesepisternum centrally impunctate. Mesepisternum and mesepimeron reaching mesocoxal cavity. Metasternum with shallow median longitudinal groove. Elytra with striae impressed and with punctures; apex rounded. Hind wings with vein r4 translucent; bear or lack wedge cell; cross vein between veins MP4 and CuA2, located at contact point between veins MP3 and MP4 (Figs 11, 18), or anterad to the contact point (Figs 29, 48). Legs with simple tarsomeres and tarsal claws. Tibial spurs present. Tarsomeres II-IV short, tarsomere V longest.

**Figures 12–16.**
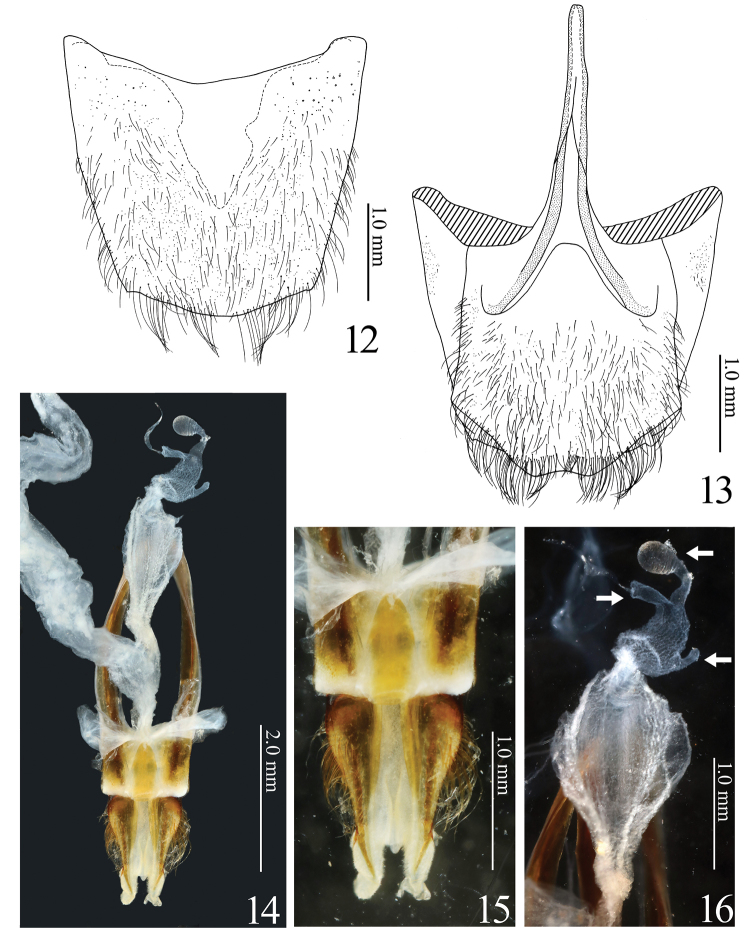
*Alaolacon candezei* Fleutiaux, 1928, female, holotype. **12** tergite VIII **13** sternite VIII, ventral view and tergite VIII, dorsal view **14** genitalia, dorsal view **15** coxites, dorsal view **16** vagina and bursa copulatrix, dorsal view (arrow: bursa copulatrix with three short sacs).

*Abdomen*. ***Male*.** Terigite VIII shorter than wide (Fig. 30) or longer than wide (Fig. 49). Sternite VIII (Figs 31, 50) wide-rectangular. Tergite IX (Figs 32, 51) wide; posterior margin notched medially. Tergite × (Figs 32, 51) semicircular. Sternite × attached to sternite IX (Figs 33, 52). ***Female*.** Tergite VIII (Fig. 12) truncate apically. Sternite VIII (Fig. 13) with spiculum ventrale robust, with apex concave or rounded.

*Genitalia*. ***Male*.** Aedeagus (Figs 34, 35, 53, 54, 57) with parameres unfused, without acute lateral subapical barb, with apical parts expanded elliptically. ***Female*.** Ovipositor (Fig. 14) stout. Coxites (Figs 15) without styli. Vagina and bursa copulatrix without sclerotized structures (Figs 16).

#### Larvae and pupae.

Unknown.

#### Distribution.

Oriental Region: India, Thailand, Vietnam, Indonesia (Sumatra, Java), Malaysia (Peninsular Malaysia, Borneo), the Philippines (Mindanao Is., Luzon Is.).

#### Bionomics.

Nothing is known of the life history and ecology.

### Alaolacon candezei

Taxon classificationAnimaliaColeopteraElateridae

Fleutiaux, 1928

Figures 1–3 , 4–11 , 12–16

Alaolacon candezei Fleutiaux, 1928: 177 (original description; type locality: Malaysia, East Malaysia (Sabah), Banggi Island).

#### Type material.

**Holotype**. Female, Banggi Island (located off the northern coast of Borneo), Sabah, Malaysia, Waterstradt leg. [MNHN] (Fig. 3). Label data: “TYPE”; [female symbol]; “Banguey/ Borneo/ Waterstradt” “= cyanipennis Cand.?/ Collection FLEUTIAUX”; “Alaolacon/ candezei/ Fleut. type/ Collection FLEUTIAUX”; “Alaolacon/ candezei Fleut./ COLLECTION FLEUTIAUX”; “Muséum paris/ Coll./ E. Fleutiaux”.

#### Diagnosis.

Body black, elytra blue and with metallic luster, legs red-black; setae white; frontal depression moderate; eye small; female antennomere III subpectinate, 1.2 × as long as wide; prothorax almost as long as wide, widest posteriorly; pronotum with anterior angles bisinuate, median longitudinal depression shallow, not reaching anterior margin or base, punctate; anterior angles of hypomeron rounded; prosternal spine inclined weakly behind procoxae; scutellum concave laterally, widest near posterior 2/5; hind wings without wedge cell, with cross vein between veins MP4 and CuA2 located at contact point between veins MP3 and MP4; female sternite VIII with apex concave.

#### Measurements.

BL: 24.0, BW: 8.35, MIE: 2.56, MAE: 3.47, OI: 74, PL: 7.64, PML: 6.67; PW: 7.70, PI: 99, EL: 15.7, EW: 8.35, EI: 188.

#### Redescription of female.

*Body* (Figs 1, 2) shiny; elytra with weak metallic luster. Color. Body black; mouth-parts brown, mandible black, galea and lacinia orange; elytra black-blue; pronotosternal sutures and legs red-black; tarsal claws yellow-brown. Hairs. Body covered with white flatted setae; antennomere I and legs with intermixed brown and white setae; antennomeres II-XI with brown setae. (Most setae of elytra lost.)

*Head*. Frontal depression moderate (Fig. 4). Eyes small. Antennomere II conical; antennomere III longest, subpectinate, 1.2 × as long as wide, 3.0 × times as long as II; apical half part of antennomere XI thinner than its basal half part (Fig. 5: dotted line). Apical maxillary palpomere 1.6 × as long as wide (Fig. 6).

*Prothorax* almost as long as wide, widest posteriorly; hind angles straight posteriorly. Pronotum with anterior angle bisinuate; median longitudinal depression shallow, not reaching anterior margin or base, punctate. Hypomeron with anterior angles rounded (Fig. 8). Prosternal spine inclined weakly (at 8 degrees) behind procoxae (Fig. 7). Scutellum (Fig. 10) 1.2 × as long as wide, concave laterally, widest near posterior 2/5. Hind wings with cross vein between veins MP4 and CuA2 apparent, not completely connected with CuA2, located at contact point between veins MP3 and MP4 (Fig. 11: arrow); wedge cell absent. Elytra widest on basal half; intervals with uniformly small punctures.

*Abdomen*. Ventrite V 0.59 × as long as wide. Tergite VIII (Fig. 12) truncate apically. Sternite VIII (Fig. 13) widest at apical 1/3, apex concave; spiculum ventrale1.4 × longer than sternite VIII.

*Genitalia* (Fig. 14). Ovipositor with coxites not sclerotized at apex (Fig. 15). Bursa copulatrix with three short sacs (Fig. 16: arrows); without sclerotized structures.

#### Male.

Unknown.

#### Distribution.

Malaysia: Sabah: Banggi Island.

#### Remarks.

**Figures 17–19.**
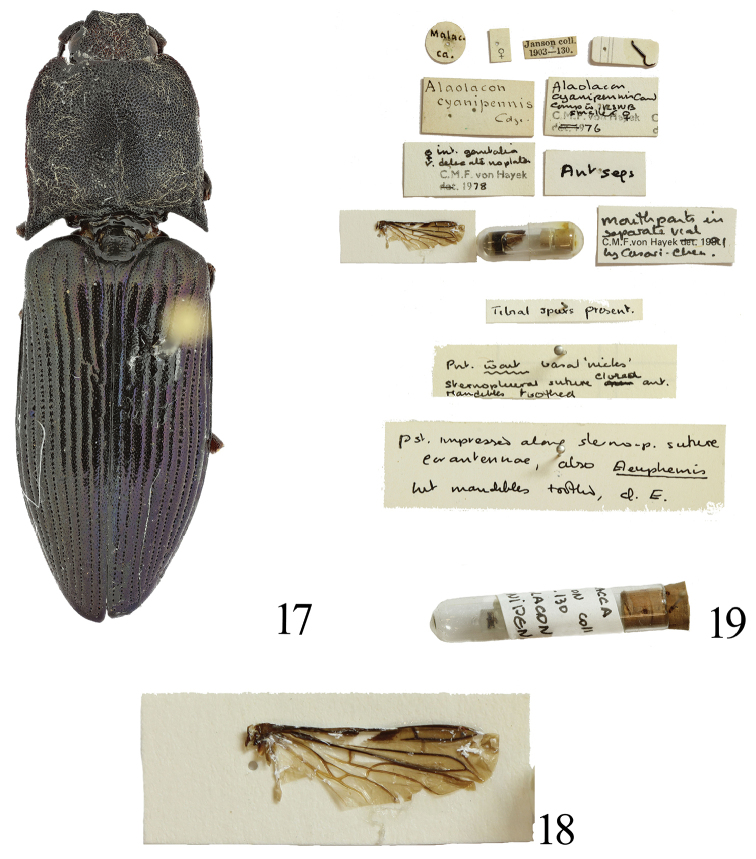
*Alaolacon cyanipennis* Candèze, 1865, female, lectotype **17** habitus, dorsal view **18** right hind wing **19** data labels and body parts.

This species is similar to *Alaolacon cyanipennis* Candèze, 1865 in large body size (24.0 mm), black body and elytra with metallic luster, but is distinguished by the following contrasting characters (*Alaolacon cyanipennis* in parentheses): female antennomere III pectinate (Fig. 5) (female antennomere III trapezoidal); prothorax widest posteriorly (Fig. 1) (prothorax widest at mid-length except for posterior angles, Fig. 17); scutellum widest near posterior 2/5 (Fig. 10) (scutellum near posterior 1/3); wedge cell of hind wings absent (Fig. 11) (wedge cell of hind wings present, Fig. 18); female sternite VIII with apex concave (Fig. 13) (female sternite VIII with apex rounded).

This species are known only from the female holotype. We predict that the males also exhibit blue elytra and with metallic luster, scutellum widest near posterior 2/5, hind wings without wedge cell and with cross vein between veins MP4 and CuA2 located at contact point between veins MP3 and MP4.

### Alaolacon cyanipennis

Taxon classificationAnimaliaColeopteraElateridae

Candèze, 1865

Figures 17–19

Alaolacon cyanipennis Candèze, 1865: 13 (original description: type locality: Peninsular Malaysia); Gemminger and Harold 1869: 1498 (catalogue of Coleoptera); Candèze 1874: 114 (monograph); Candèze 1891: 29 (catalogue; description of type locality: Malacca); Schwarz 1906: 316 (catalogue); Hyslop 1921: 625 (type species); Schenkling 1925: 40 (catalogue); Fleutiaux 1926: 102 (catalogue); Casari-Chen 1993: (description; designation of homeotype); Suzuki 2004: 152 (record from Sumatra).

#### Type material.

**Lectotype**. Female, Malacca, West Malaysia (Peninsular Malaysia), Malaysia, Janson coll. [BMNH] (Fig. 19). Label data: “Malacca”; [female symbol]; Janson coll/ 1903-130.; “Alaolacon/ cyanipennis/ Cdz. ”; “Alaolacon/ cyanipennis Cand./ Comp to RSNB/ smaller female/ C.M.F. von Hayek/ 1976”; “female int. genitalia/ delicate no plates/ C.M.F. von Hayek/ 1978”; “Antseps”; “mouthparts in/ separate vial/ C.M.F. von Hayek 1991/ by Casari-Chen”; and with the authors’ red lectotype label: “LECTOTYPE/ Alaolacon cyanipennis/ Candèze, 1865”.

#### Diagnosis.

Body black, elytra blue-black and with metallic luster; setae white; female antennomere III trapezoidal, 1.4 × as long as wide; prothorax as long as wide, widest at mid-length except for posterior angles; pronotal anterior angles bisinuate and rounded; anterior angles of hypomeron rounded; prosternal spine inclined weakly behind procoxae; scutellum concave laterally, widest near posterior 1/3; hind wings with wedge cell, with cross vein between veins MP4 and CuA2 located at contact point between veins MP3 and MP4; female sternite VIII with rounded apex.

#### Description.

See Casari-Chen (1993) for a detailed description.

#### Distribution.

Malaysia: the Peninsular Malaysia. Indonesia: Sumatra.

#### Remarks.

Candèze (1865) did not provide the number of the type specimens. Candèze (1865) mentioned that “*Elle a été découverte et apportée récemment en Europe par M. de Castelnau. Je l’ai vue dans sa collection, ainsi que dans celle de M. le comte de Mniszech*”. Mniszech’s collection went to Laporte de Castelnau, part of this went to Janson and then to BMNH. Candèze’s first collection of Elateridae (up to 1869) went to the BMNH, while a second collection of Elateridae went to IRSNB (Bousquet 2016). BMNH can be most expected to hold types of this species because it was described before 1869. Label data of the examined specimen in BMNH agree with the original description. The external features of the specimen also agree with the original description. Thus, the specimen should be considered a syntype. Casari-Chen (1993) considered the type specimen as a homeotype. We designated the known syntype as lectotype to stabilize the classification.

We could not locate other syntypes including at IRSNB in this time. Laporte de Castelnau’s first collection was given to the National Institution of the Promotion of Science in Washington DC but was probably destroyed by fire, while part of his later collection was left to the Melbourne Museum in Australia (Bousquet 2016).

Only female specimens are known (Candèze 1865; Suzuki 2004). Only this species exhibits hind wings with wedge cell in this genus, whereas the other species lost wedge cell of hind wings. We predict that the male could also be recognized by presence of the wedge cell.

### Alaolacon fujiokai sp. n.

Taxon classificationAnimaliaColeopteraElateridae

http://zoobank.org/FF52714A-2F8B-413C-9777-C96D283C3465

Figures 20–21 , 22–29 , 30–35 , 36–37

#### Etymology.

The name of this species honors Mr. Masahiro Fujioka for providing the material.

#### Type material.

**Holotype**. Male, Tawau, East Malaysia (Sabah), Malaysia, V 2014 [ELKU].

#### Diagnosis.

Body black, elytra blue and with metallic luster, legs black; setae black on dorsal side and white on ventral side; frontal depression deep; eye small; prothorax almost as long as wide, widest posteriorly; pronotum with anterior angles bisinuate and rounded, medina longitudinal depression deep, extending from before pronotal anterior margin to base, punctate; prosternal process inclined strongly behind procoxae; anterior angles of hypomeron acute; scutellum concave laterally, widest near posterior 1/3; hind wings with cross vein between veins MP4 and CuA2 located anterad to contact point between veins MP3 and MP4, without wedge cell; median lobe of male aedeagus stout.

#### Measurements.

BL: 18.9, BW: 6.11, MIE: 2.08, MAE: 3.05, OI: 68, PL: 5.95, PML: 5.23; PW: 5.91, PI: 101, EL: 12.1, EW: 6.11, EI: 197.

#### Description of male.

**Figures 20, 21.**
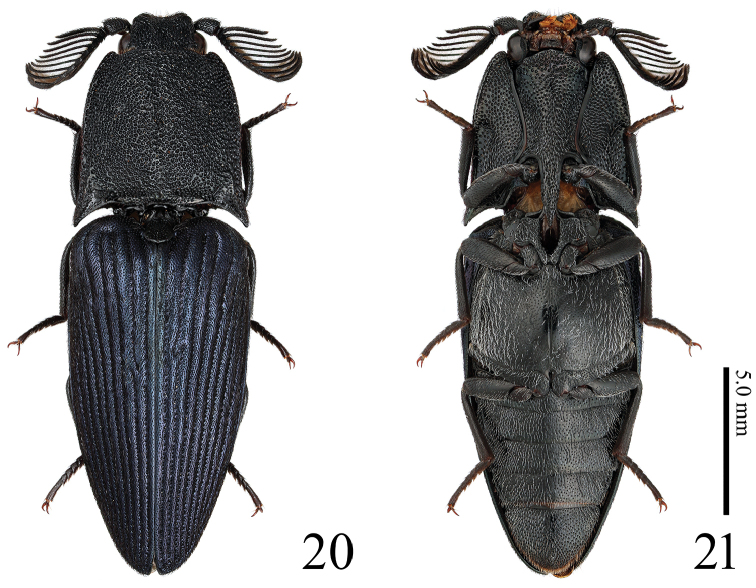
*Alaolacon fujiokai* sp. n., male, holotype. **20** habitus, dorsal view **21** ditto, ventral view.

*Body* (Figs 20, 21) shiny, elytra with metallic luster. Color. Black except for elytra black-blue; mouth-parts brown-black, but mandible black, galea and lacinia orange; apical edge of tarsal segment V and tarsal claws red-brown; pregenital segments and aedeagus black-brown. Hairs. Dorsal surface covered with black flatted setae; ventral surface with white flatted setae; legs with intermixed black and white setae; mouth-parts and pronotal anterior margin near eyes with yellow-brown setae; filiform setae of antennomeres III-XII brown and long.

**Figures 22–29.**
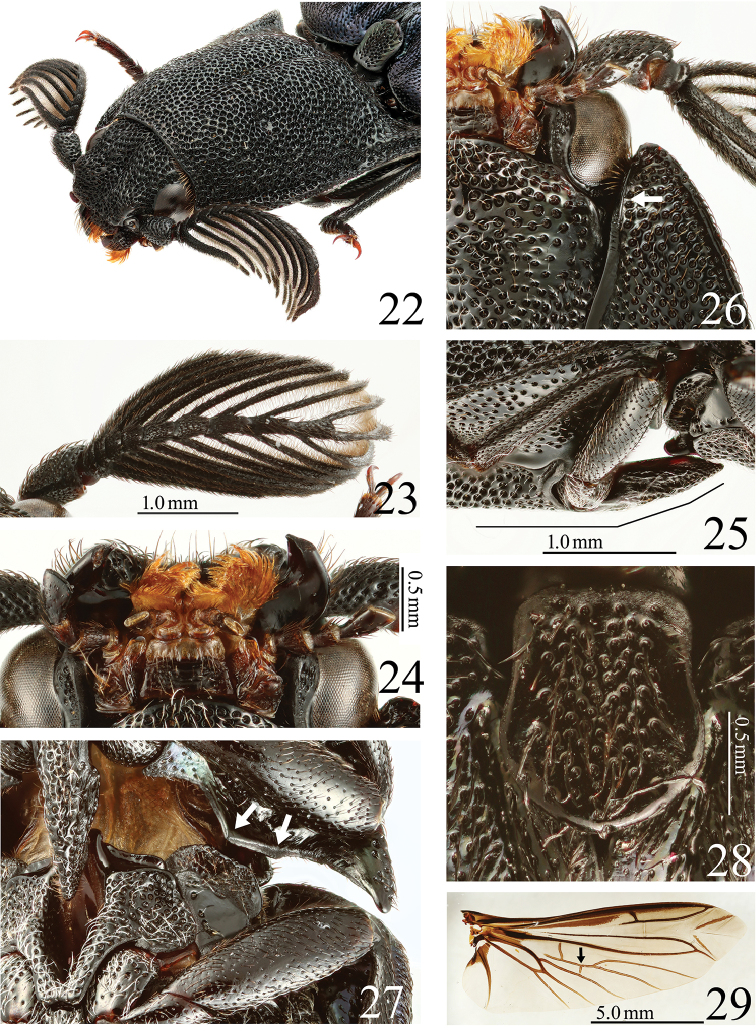
*Alaolacon fujiokai* sp. n., male, holotype. **22** head and pronotum, anterolateral view **23** right antenna, dorsal view **24** mouth-parts **25** prosternal process, lateral view **26** anterior angle of hypomeron (arrow: mesal edge carinate anterolaterad) **27** posterior part of hypomeron and mesothorax, ventral view (arrows: posterior margin with two angles) **28** scutellum **29** right hind wing (arrow: cross vein between veins MP4 and CuA2 located anterad to contact point between veins MP3 and MP4).

*Head* (Fig. 22). Frontal depression deep. Eyes small. Antennomere I long; antennomere II short, dish-shaped; antennomeres III-XI flabellation strong; antennomere XII elongate (Fig. 23). Apical maxillary palpomere 1.8 × as long as wide (Fig. 24) (Mandibles chipped in apical parts.)

*Prothorax* almost as long as wide, widest posteriorly; sides rounded anteriorly, liner posteriorly. Pronotum with anterior angles bisinuate and rounded; median longitudinal depression deep, extending from before pronotal anterior margin to base, punctate. Prosternal spine inclined strongly (at 18 degrees) behind procoxae (Fig. 25). Hypomeron with anterior angles acute (Fig. 26). Scutellum (Fig. 28) concave laterally, 1.2 × as long as wide, widest near posterior 1/3. Hind wings with cross vein between veins MP4 and CuA2 apparent, not completely connected with CuA2, located anterad to contact point between veins MP3 and MP4 (Fig. 29: arrow); wedge cell absent. Elytra with sides almost parallel on basal half; intervals with small and coarse punctures.

**Figures 30–35.**
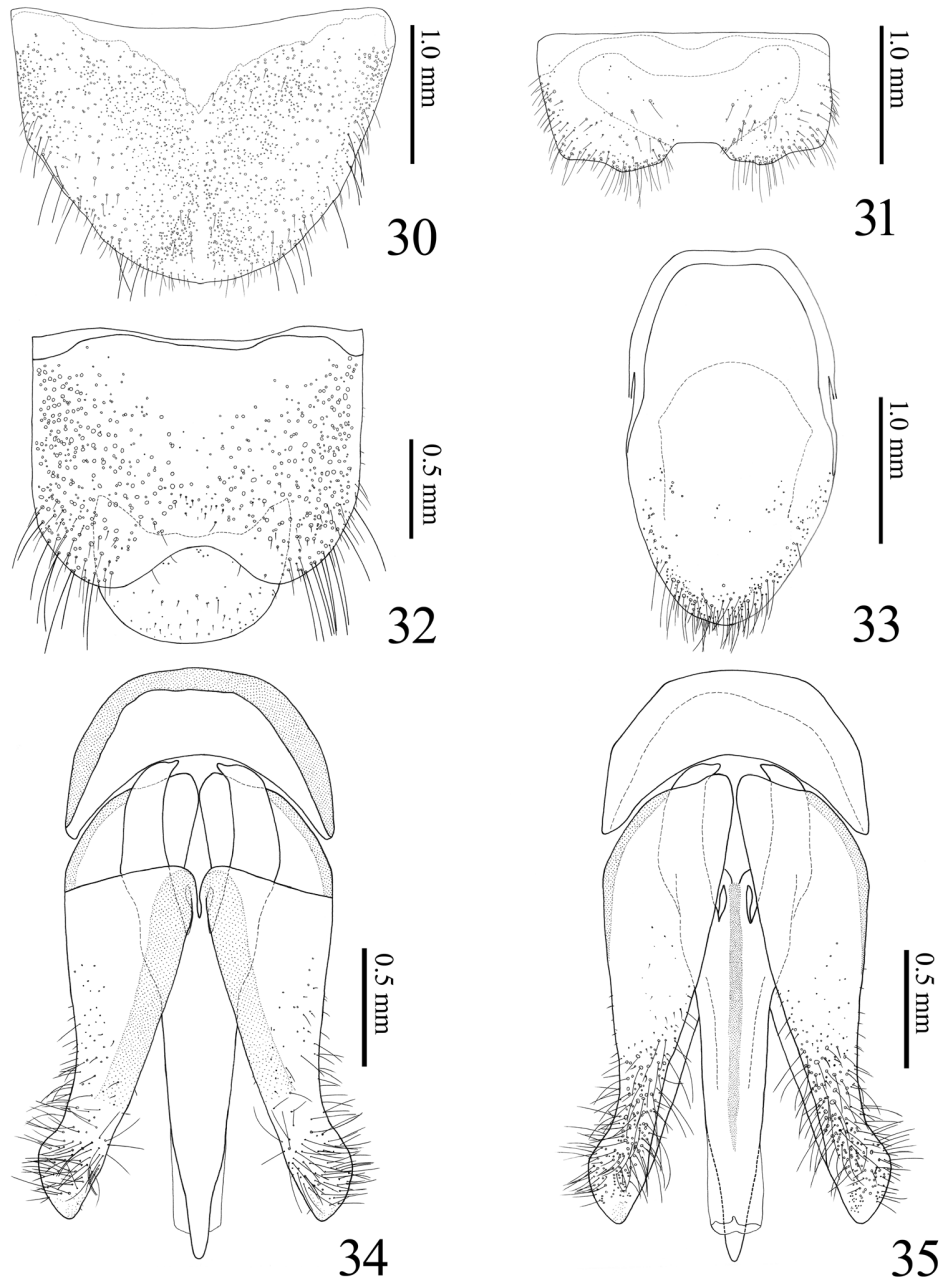
*Alaolacon fujiokai* sp. n., male, holotype. **30** tergite VIII **31** sternite VIII **32** tergites IX–X **33** sternites IX–X **34** aedeagus, dorsal view **35** ditto, ventral view.

*Abdomen*. Ventrite V 0.67 × times as long as wide. Tergite VIII (Fig. 30) 0.72 × as long as wide, colorless basal area. Sternite VIII (Fig. 31) with darker W-shaped band; median notch shallow and truncate transversally. Tergite IX (Fig. 32) with median notch shallow and rounded. Sternite IX (Fig. 33) narrowed abruptly on posterior half to apex. Aedeagus (Figs 34, 35). Median lobe stout; basal struts 0.35 × total length of median lobe. Parameres with dense and long setae. Basal piece 0.29 × total length of aedeagus.

**Figures 36, 37.**
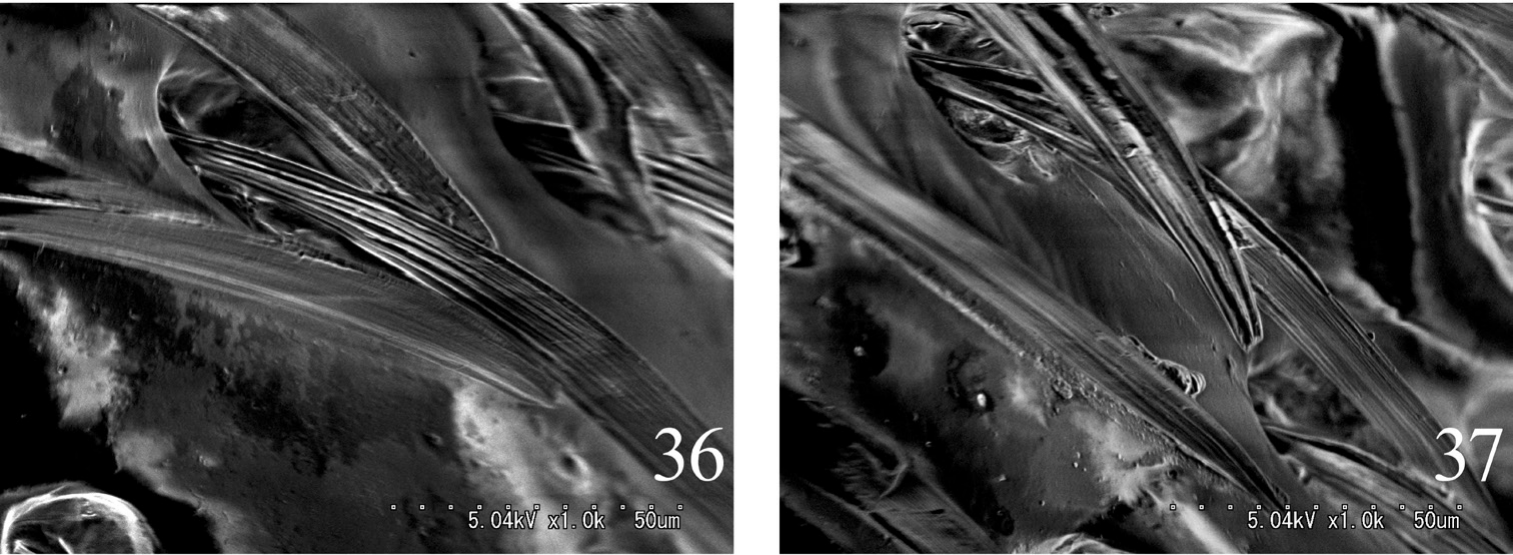
*Alaolacon fujiokai* sp. n., male, holotype, setae of elytra. **36** median part **37** apical part.

#### Female.

Unknown.

#### Distribution.

Malaysia: Sabah: Tawau.

#### Remarks.

This species is distinct by black body, blue elytra with metallic luster, black setae on dorsal side, white setae on ventral side and strong antennomeres III-XI flabellation. It is similar to *Alaolacon candezei* Fleutiaux, 1928 in having a black body, blue elytra with metallic luster, pronotum anterior angles bisinuate and rounded, and scutellum concave laterally, except for drastic sexual differences of antennae, but is distinguished by the following contrasting characters (*Alaolacon candezei* in parentheses): legs black (Fig. 21) (legs red, Fig. 2); setae black on dorsal side and white on ventral side (Fig. 20) (all setae white, Fig. 1); frontal depression deep (Fig. 22) (frontal depression moderate, Fig. 4); pronotal median longitudinal depression extending from before pronotal anterior margin to base (Fig. 22) (pronotal median longitudinal depression not reaching anterior margin or base, Fig. 4); anterior angles of hypomeron acute (Fig. 26) (anterior angles of hypomeron rounded, Fig. 8); prosternal spin inclined strongly behind procoxae (Fig. 25) (prosternal spine inclined weakly behind procoxae, Fig. 7); scutellum widest near posterior 1/3 (Fig. 28) (scutellum widest near posterior 2/5, Fig. 10); hind wings with cross vein between veins MP4 and CuA2 located anterad to contact point between veins MP3 and MP4 (Fig. 29: arrow) (hind wings with cross vein between veins MP4 and CuA2 located at contact point between veins MP3 and MP4, Fig. 11: arrow).

*Alaolacon fujiokai* and *Alaolacon candezei* are similar species from the same island, but we recognized they are different species by the setal color and the hind wing venation. We believe that setal complementary color difference probably is not caused by sexual dimorphism because such dimorphism has not previously been observed in species of the Agrypninae. We also believe that differences in hind wing venation are unlikely to be caused by sexual dimorphism because such dimorphism has not previously been observed in species with flying females.

### Alaolacon megalopus sp. n.

Taxon classificationAnimaliaColeopteraElateridae

http://zoobank.org/83188584-DF58-41F6-BF06-BF6702F909A8

Figures 38–40 , 41–48 , 49–54

Eumoeus murrayi Candèze, 1874; Fleutiaux 1928: 178 (mention a specimen from Java at IRSNB); Casari-Chen 1993: 241 (examined a male specimen from Java). Misidentification.

#### Etymology.

A combination of the Greek *megalos*, meaning great, and the Greek *ops*, meaning eye, refer to the large compound eyes.

#### Type material.

**Holotype**. Male, Java, Indonesia [IRSNB] (Fig. 40).

#### Diagnosis.

Body brown, without metallic luster; setae yellow-brown; frontal depression moderate; eye very large; prothorax wider than long, widest posteriorly; pronotal anterior angles rounded; median longitudinal depression shallow, located at anterior half, punctate; anterior angles of hypomeron acute; prosternal spine inclined strongly behind procoxae; scutellum 1.5 × as long as wide; sides of scutellum parallel; hind wings with cross vein between veins MP4 and CuA2 located just anterior to contact point between veins MP3 and MP4, without wedge cell; male tergite VIII longer than wide; median lobe of male aedeagus elongate.

#### Measurements.

BL: 11.8, BW: 3.54, MIE: 1.02, MAE: 2.31, OI: 44, PL: 3.11, PML: 2.74, PW: 3.26, PI: 95, EL: 7.70, EW: 3.54, EI: 218.

#### Description.

**Figures 38–40.**
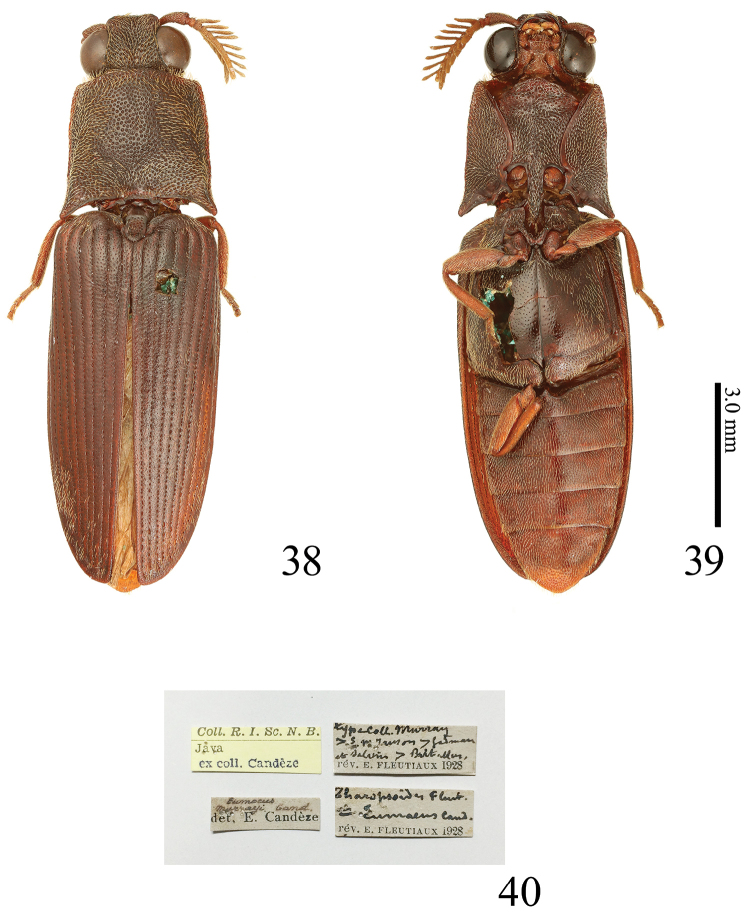
*Alaolacon megalopus* sp. n., male, holotype. **38** habitus, dorsal view **39** ditto, ventral view **40** data labels.

*Body* (Figs 38, 39) shining, without metallic luster. Color. Body brown; antennomere I, pronotal lateral margin, elytra, legs, abdomen paler; antennomeres II-XII, mouth-parts, pregenital segments and aedeagus yellow-brown, but mandible brown. Hairs. Body covered with yellow-brown setae; antennomeres III-XII with short filiform setae at ventral surface.

**Figures 41–48.**
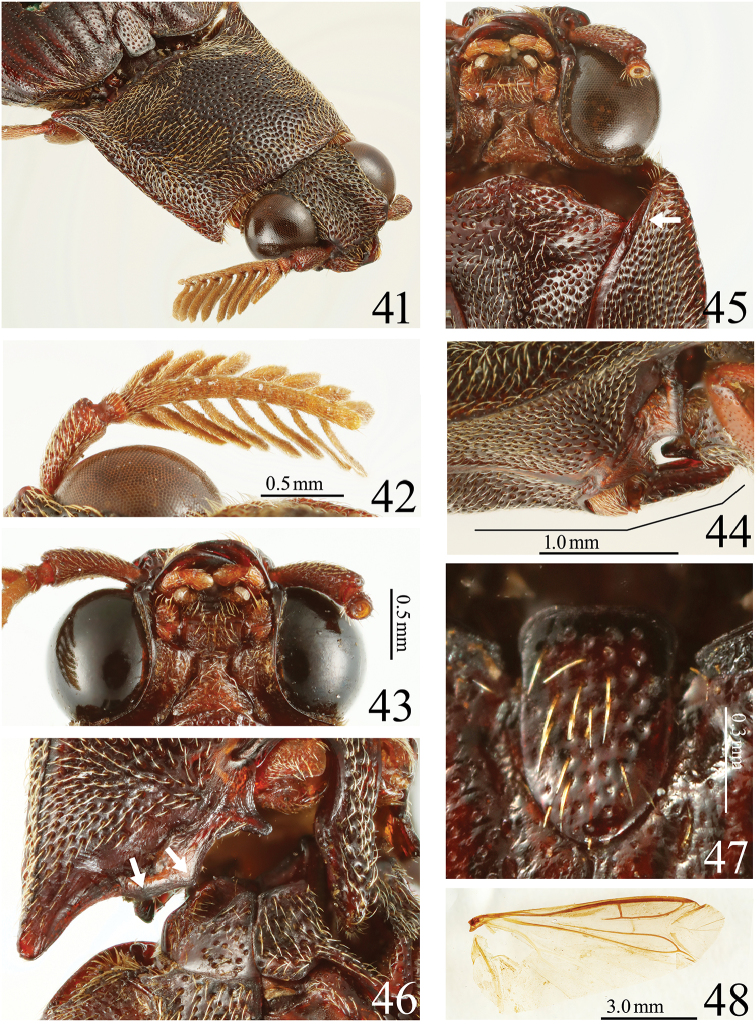
*Alaolacon megalopus* sp. n., male, holotype. **41** head and pronotum, anterolateral view **42** right antenna, dorsal view **43** mouth-parts **44** prosternal process, lateral view **45** anterior angle of hypomeron (arrow: mesal edge carinate anterolaterad) **46** posterior part of hypomeron and mesothorax, ventral view (arrows: posterior margin with two angles) **47** scutellum **48** right hind wing.

*Head* (Fig. 41). Frontal depressed moderate. Eyes very large. Antennomere I elongate; antennomere II short and obconical; antennomeres III-X flabellation moderate (Fig. 42). Apical maxillary palpomere (Fig. 43) rounded, 1.3 × as long as wide. (Antennomeres XI-XII of right antenna and antennomeres III-XII of left antenna lost.)

*Prothorax* wider than long; sides widest posteriorly, rounded anteriorly, liner posteriorly. Pronotum convex; anterior angles rounded; median longitudinal depression shallow, located at anterior half, punctate (Fig. 41); central area with two shallow concaves. Prosternal spine with lateral margin of dorsal side expanded laterally, inclined strongly (at 15 degrees) behind procoxae (Fig. 44). Hypomeron with anterior angles acute (Fig. 45); punctures more homogeneous than prosternal punctures in density and size. Scutellum (Fig. 47) 1.5 × as long as than wide; sides parallel. Elytra with sides almost parallel on basal half; intervals with small and coarse punctures. Hind wings (Fig. 48) with veins posterior to MP3 translucent; cross vein between veins MP4 and CuA2 not completely connected with CuA2, located just anterior to contact point between veins MP3 and MP4; wedge cell absent (Fore legs except for coxae, tarsomeres IV-V and claw of right middle leg, tarsomere V and claw of left middle leg, tarsi and claw of right hind leg, and left hind leg lost.)

**Figures 49–54.**
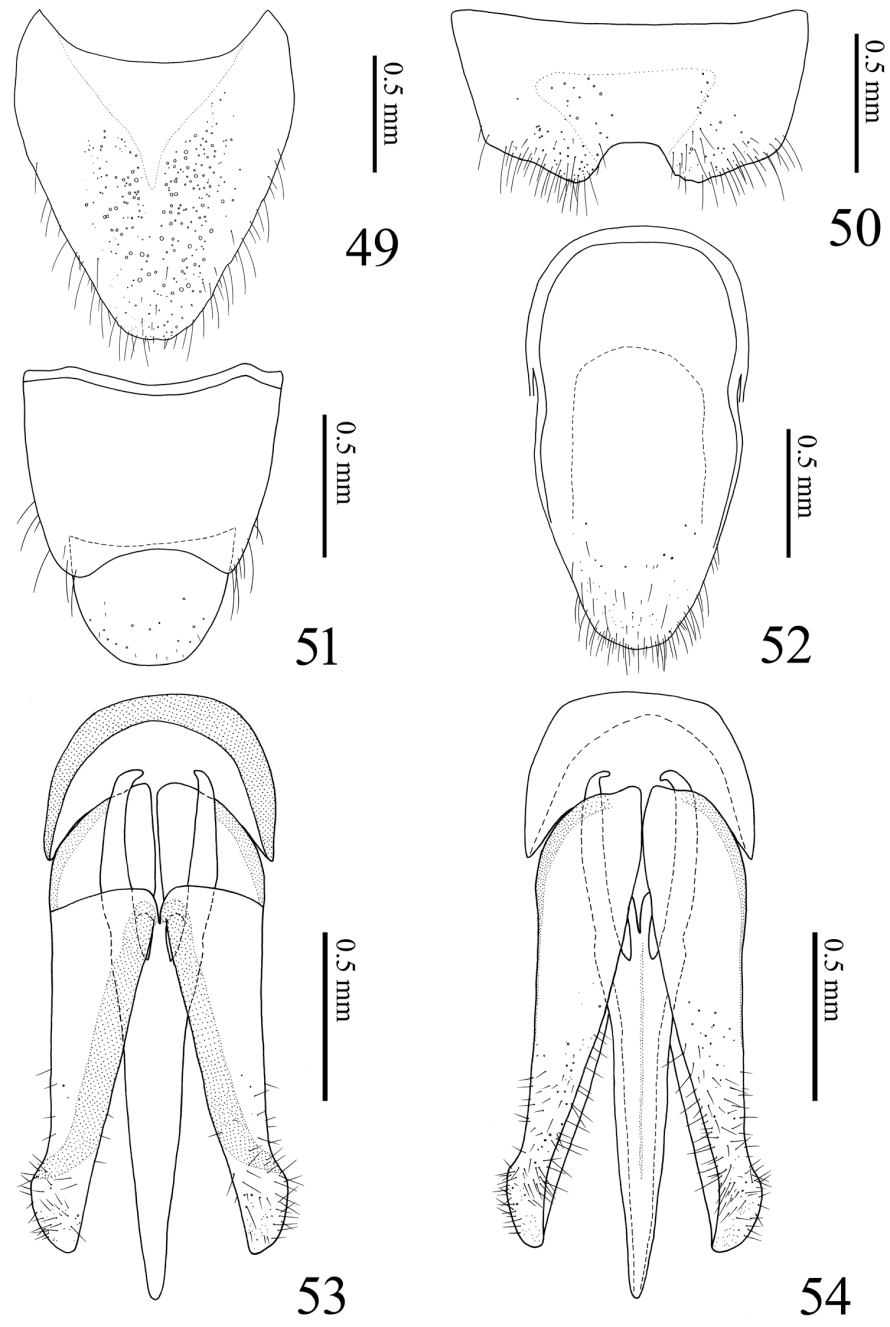
*Alaolacon megalopus* sp. n., male, holotype. **49** tergite VIII **50** sternite VIII **51** tergites IX–X **52** sternites IX–X **53** aedeagus, dorsal view **54** aedeagus, ventral view.

*Abdomen*. Ventrite V 0.65 × as long as wide. Tergite VIII (Fig. 49) 1.2 × as long as wide; basal area translucent. Sternite VIII (Fig. 50) with central area translucent; median notch shallow and truncate transversally. Tergite IX (Fig. 51) with median notch shallow and rounded. Tergite × (Fig. 51) posterior margin rounded. Sternite IX (Fig. 52) wide; posterior half abruptly narrowed to apex; posterior margin rounded. Aedeagus (Figs 53, 54). Median lobe elongate, basal struts 0.37 × total length of median lobe. Parameres with sparse and short setae. Basal piece 0.28 × total length of aedeagus.

#### Female.

Unknown.

#### Distribution.

Indonesia: Java.

#### Remarks.

The holotype is damaged with most appendages lost. The holotype of this species was identified as *Eumoeus murrayi* (= *Alaolacon murrayi* comb. n.) by Candèze (Fleutiaux, 1928), but separated from *Alaolacon murrayi* by the following characteristics (the holotype of *Alaolacon murrayi* in parentheses): eye very large (OI: 44) (eye large, OI: 50); anterior angles of pronotum rounded (Fig. 38) (anterior angles of pronotum bisinuate, Fig. 56); pronotal median longitudinal depression shallow, located at pronotal anterior half and punctate (Fig. 41) (pronotal median longitudinal depression not reaching anterior margin or base and impunctate at posterior half); scutellum 1.5 × as long as wide (Fig. 47) (scutellum 1.3 × as long as wide, Fig. 56); scutellum sides parallel (Fig. 47) (scutellum sides concave and widest posteriorly, Fig. 56); hind wings with cross vein between veins MP4 and CuA2 (Fig. 48) (hind wings without cross vein between veins MP4 and CuA2); male tergite VIII longer than wide (Fig. 49) (male tergite VIII shorter than wide).

**Figures 55–58.**
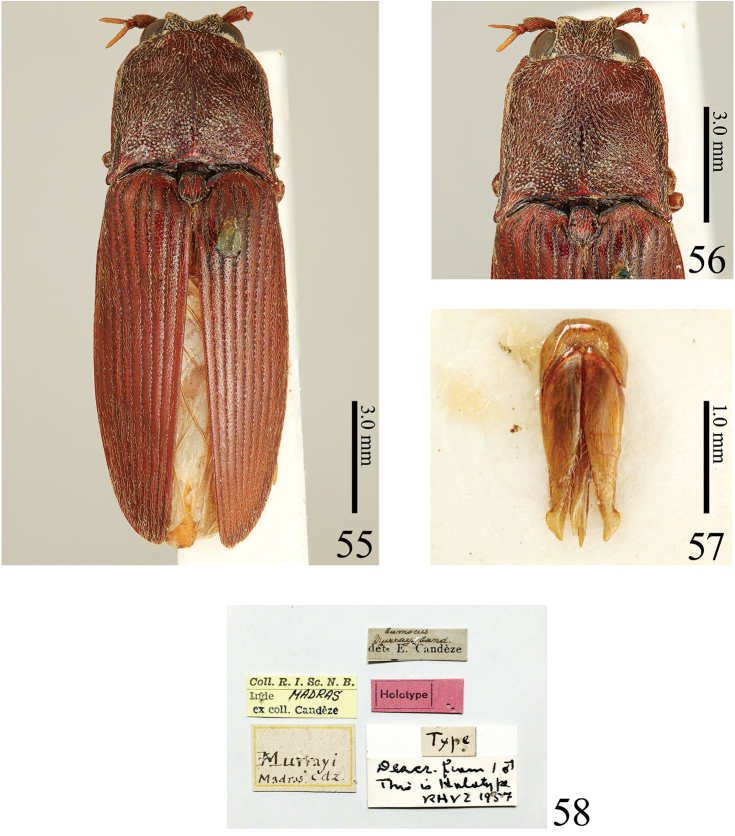
*Alaolacon murrayi* (Candèze, 1874), comb. n., male, holotype. **55** habitus, dorsal view **56** head, pronotum and scutellum **57** aedeagus, ventral view **58** data labels.

Only this species exhibits parallel sides of scutellum in this genus. The scutellum shape could be a useful specific diagnostic feature for this species including its female.

### Alaolacon murrayi

Taxon classificationAnimaliaColeopteraElateridae

(Candèze, 1874) comb. n.

Figures 55–58

Eumoeus murrayi Candèze, 1874: 113 (original description on male; type locality: Madras, India), 214 (as “Eumaeus murrayi”; index); Schwarz 1906: 40 (catalogue); Hyslop 1921: 645 (type species); Schenkling 1925: 51 (as “Eumaeus”; catalogue); Fleutiaux 1928: 178 (comments); Casari-Chen 1993: 241 (description on male; examination of holotype; misspelled Eumaeus murrayi); Casari 2008: 158 (morphological phylogeny of Hemirhipini genera; misspelled Eumaeus murrayi), 161 (drawing of habitus).
Tharopsides harmandi Fleutiaux, 1918: 235 (original description on male; type locality: Bangkok, Thailand); Fleutiaux 1924: 177 (republish of original description); Schenkling 1927: 509 (catalogue of world Elateridae); Fleutiaux 1940: 40 (record of male from Vietnam); Fleutiaux 1947: 307 (as synonymy of Luzonicus murrayi (Candèze, 1874)).
Luzonicus murrayi (Candèze, 1874): Fleutiaux 1947: 307 (change generic status; description).

#### Type material.

**Holotype.** Male, Madras, India, Murray leg. [IRSNB] (Fig. 58).

#### Diagnosis.

Body red-brown, without metallic luster; setae yellow-brown; frontal depression deep; eye large; prothorax shorter than wide, widest posteriorly; pronotum with anterior angles bisinuate, median longitudinal depression not reaching anterior margin or base and impunctate at posterior half; anterior angles of hypomeron acute; prosternal spine inclined strongly behind procoxae; scutellum 1.3 × as long as wide, with sides straight, widest posteriorly; hind wings without cross vein between veins MP4 and CuA2 and wedge cell; male tergite VIII shorter than wide; median lobe of male aedeagus elongate.

#### Measurements.

BL: 14.9, BW: 4.85, MIE: 1.43, MAE: 2.85, OI: 50, PL: 4.34, PML: 3.68, PW: 4.64, PI: 94, EL: 10.1, EW: 4.85, EI: 208.

#### Description.

See Casari-Chen (1993) for a detailed description.

#### Distribution.

India. Thailand. Vietnam.

#### Remarks.

This species is only known from the male.

## Discussion

Candèze (1874) produced a misspelling of *Eumoeus*, writing “EUMÆUS” in the index on page 214. Candèze (1891) used “EUŒUS” on page 29 and “Eumœus” in index on page 243. This means that Candèze (1891) had already recognized *Eumoeus* as a valid name. However, Fleutiaux (1940) considered *Eumoeus* as a wrong spelling of *Eumaeus* and used *Eumaeus* as the valid name. He used *Tharopsides* Fleutiaux, 1918 as the replacement name for “*Eumaeus* Candèze, 1874” because it became a junior homonym for the genus *Eumaeus* Hubner, 1816 of Lepidoptera.

*Eumoeus* and *Tharopsides* were described from males exhibiting 12-segmented and biflabellate antennae, whereas *Luzonicus* were described from female exhibiting 11-segmented and filiform to subpectinate antennae. Fleutiaux (1947) inferred that there was an occurrence of sexually dimorphic antennae of these genera, and that *Luzonicus* was therefore the senior synonym for *Eumoeus* and *Tharopsides*. Actually *Eumoeus* is the senior synonym for both *Luzonicus* and *Tharopsides* because the actions of Fleutiaux (1940) are nullified.

*Alaolacon* Candèze, 1865 was only known from female with 11-segmented and pectinate antennae. We determined that a male specimen (the holotype of *Alaolacon fujiokai* sp. n.), in possessing biflabellate antennae, belongs to *Alaolacon* because of the similarity to *Alaolacon cyanipennis* and *Alaolacon candezei* including: black body, blue elytra with metallic luster, pronotum anterior angles bisinuate, scutellum concave laterally. This association demonstrates that *Alaolacon* also has sexually dimorphic antennae.

In the tribe Hemirhipini, only four genera, *Alaolacon*, *Eumoeus*, *Mocquerysia* Fleutiaux, 1899 and *Eleuphemus* Hyslop, 1921 have strongly sexually dimorphic antennae. Their males exhibit 12-segmented and biflabellate antenna, and females exhibit 11-segmented and subpectinate antennae. *Eleuphemus* is separated from *Alaolacon*, *Eumoeus*, *Mocquerysia* (the latter three genera in parentheses) by the supra-antennal carinae continuous across frons (supra-antennal carina not continuous across frons) and mandible without subapical tooth (mandible with subapical tooth). *Mocquerysia* is separated from *Alaolacon* and *Eumoeus* (the latter two genera in parentheses), prosternal suture shortly grooved (prosternal suture not grooved), scutellum narrowed apically and with straight side (scutellum widest apically and concave laterally or with parallel sides in *Alaolacon megalopus* sp. n.), elytral intervals flat (elytral intervals convex).

Candèze (1874) suggested that *Alaolacon* should be combined with *Eumoeus* if its male had biflabellate antennae. We recognized that *Alaolacon* and *Eumoeus* are similar by many structures: setae flat, wider at midlength than base, with longitudinal micro carinae (Figs 36, 37); interspaces greater than puncture diameter except for smaller on head and pronotum; hypomeron mesal edge carinate anterolaterad (Figs 8, 26, 45: arrow); hind wings with vein r4 translucent (Figs 11, 18, 29, 48). The two genera could not be separated from each other except by antennal morphology. This non-antennal similarity suggests that the two genera should be considered synonyms because antennal morphology is dimorphic in several other Elateridae. We propose that the two genera should be considered synonyms. Accordingly, the priorities of the generic names are following: *Tharopsides* < *Luzonicus* < *Eumoeus* < *Alaolacon*.

*Luzonicus bakeri* Fleutiaux, 1916 and *Tharopsides bakeri* Fleutiaux, 1918 are eventual homonyms since *Luzonicus* and *Tharopsides* are junior synonyms of *Alaolacon*. We propose *Alaolacon philippinensis* nom. n., as the replacement name for *Alaolacon bakeri* (Fleutiaux, 1916) comb. n. *Alaolacon* currently contains eight species, 1, *Alaolacon bakeri*, 2, *Alaolacon candezei*, 3, *Alaolacon cyanipennis*, 4, *Alaolacon fujiokai*, 5, *Alaolacon griseus* Candèze, 1874, 6, *Alaolacon megalopus*, 7, *Alaolacon murrayi* and 8, *Alaolacon philippinensis*.

We could not find the types of two species, *Alaolacon bakeri* and *Alaolacon philippinensis*, and have not examined these species. Further effort to find the localities of the types of the two species are needed in order to understand the complete picture of these species.

### Key to species for adults of the genus *Alaolacon*

**Table:** 

1	Head and pronotum brown to red-brown	**2**
–	Head and pronotum black	**4**
2	Prothorax longer than wide, elytra red-brown but brown-black on posterior half	***Alaolacon bakeri* (Fleutiaux, 1916)**
–	Prothorax shorter than wide, elytra brown to red-brown	**3**
3	Scutellum widest posteriorly	***Alaolacon murrayi* (Candèze, 1874) comb. n.**
–	Scutellum with parallel sides	***Alaolacon megalopus* sp. n.**
4	Elytra blue or blue-black, and with metallic luster	**5**
–	Elytra black and without metallic luster	**7**
5	Setae black dorsally and white ventrally	***Alaolacon fujiokai* sp. n.**
–	All setae white	**6**
6	Prothorax widest posteriorly, wedge cell of hind wings absent	***Alaolacon candezei* Fleutiaux, 1928**
–	Prothorax widest at mid-length except for posterior angles, wedge cell of hind wings present	***Alaolacon cyanipennis* Candèze, 1865**
7	Ventral surface red-brown	***Alaolacon griseus* Candèze, 1874**
–	Ventral surface black	***Alaolacon philippinensis* nom. n.**

## Supplementary Material

XML Treatment for Alaolacon

XML Treatment for Alaolacon candezei

XML Treatment for Alaolacon cyanipennis

XML Treatment for Alaolacon fujiokai

XML Treatment for Alaolacon megalopus

XML Treatment for Alaolacon murrayi
